# Statistics for undergraduate medical students in Sudan: associated factors for using statistical analysis software and attitude toward statistics among undergraduate medical students in Sudan

**DOI:** 10.1186/s12909-022-03960-0

**Published:** 2022-12-22

**Authors:** Elfatih A. Hasabo, Ghassan E. Mustafa Ahmed, Raed M. Alkhalifa, Mai D. Mahmoud, Sara Emad, Roaa B. Albashir, Mohamed E. Mansour, Elmuiz A. Hsabo

**Affiliations:** 1 Faculty of Medicine, University of Khartoum, Khartoum, Sudan; 2 Faculty of Medicine, Red Sea University, Port Sudan, Sudan; 3 Faculty of Medicine, University of El-Imam El-Mahdi, Kosti, Sudan; 4 Department of Urology, Manchester University NHS Foundation Trust, Wythenshawe Hospital, Manchester, UK

**Keywords:** Statistics, Undergraduate medical students, Attitude, Associated factors, Sudan, Statistical analysis software

## Abstract

**Introduction:**

Statistics helps medical students understand research. Without understanding statistics, students can’t choose the proper analysis in their research. We aimed to assess the attitude toward statistics, usage of statical software and associated factors for using statistical analysis software in Sudan.

**Method:**

A cross-sectional online survey was distributed among undergraduate medical students across ten Sudanese universities. The study aimed to measure their attitude towards statistics using Survey of Attitudes Toward Statistics (SATS-36) scale.

**Results:**

In total, 489 students were participated with a mean age of 21.94 ± 1.61 and a slight female preponderance (52%, *n* = 256). The overall attitude towards statistics was 4.64 ± 0.91. The mean attitude scores for the components of SATS-36 scale was higher for students who were using statistical analysis software demonstrating significant difference in affect (*p* = 0.002), cognitive competence (*p* = 0.002), value (*p* = 0.002), Interest (*p* = 0.004) and Effort (*p* = 0.029). Almost half of the students (47%) had attended a biostatistics workshop with only 26% of them reported using statistical analysis software. Of the latter group, 72% (*n* = 91) used SPSS while 50% (*n* = 64) used excel. Univariate logistic regression showed students who had previously used an statistical software were more likely to be studying in their sixth year compared with second year (OR: 12.652, CI 95% 4.803– 33.332; *p* < 0.001), older age (OR: 1.224, CI 95% 1.079– 1.388; *p* = 0.002), attended a course in research methodology (OR: 3.383, CI 95% 2.120– 5.398; *p* < 0.001) or biostatistics (OR: 1.886, CI 95% 1.252– 2.841; *p* = 0.002), initiated or participated in a research project (OR:4.349, CI 95% 2.839 – 6.661;*p* < 0.001) or published a paper (OR: 8.271, CI 95% 3.542 – 19.312; *p* < 0.001).

**Conclusions:**

The study showed an average attitude towards statistics among medical students. Being at higher years, participating or publishing research and attending research workshop are associated with the usage of statistical software. Also, few students were using statistical software.

## Introduction

Biostatistics is one of the branches of applied statistics concerned with the application of statistical knowledge in research related to health [1]. The understanding and interpretation of health-related data depend mainly on the knowledge of statistical methods. Because of the vital role of statistics in the medical field, it has been taught at the undergraduate level in most medical schools [2].

Understanding and learning medical statistics is essential for undergraduate medical students during data analysis and designing research [3]. Without a good knowledge of statistical methods, medical students can’t improve their skills in evaluating published research and applying the evidence-based practice in the future [4–6]. Biostatistics gives the medical student and the future physician the capacity for critical thinking and rational decision-making to choose the best options that suit the patients [7].

Understanding medical students' attitudes towards statistics is crucial as a positive attitude can reflect a good future medical practice that is evidence-based, and a negative attitude can reflect the need for further emphasis on the importance of statistics and its applications. Statistical analysis software helps researchers to transform their raw data into tables, graphs and figures [8]. Researchers were using different statistical software to assist them in analyzing their data. Investigating the students' attitudes towards statistics and their usage of the different statistical software would help in improving the quality of medical education.

No previous data or researches were published about the attitude of medical students toward statistics and the usage of statistical analysis software in Sudan. In this study, we aimed to assess the medical students’ attitude toward statistics, usage of statical analysis software and associated factors for using statistical analysis software among undergraduate medical students in Sudan.

## Methods

### Study design and settings

This cross-sectional online survey was conducted among undergraduate medical students studying at ten Sudanese public universities, five of which are based within the capital of Sudan, Khartoum. All participating universities are exclusively government-funded. The data collection took place from 1^st^ of November 2020 till 31^st^ of January 2021. The setting for this study was similar to another study published previously in Sudan which examined the same target population and we used their methods in our study [9].

Teaching statistics course across included universities is similar, starting from second or third year for all undergraduate medical students. Students were taught statistics such as measures of central tendency, test of significant difference, measures of association, linear and logistic regression…etc., and they are been examined at the end of course.

### Participants

All medical students studying in their second year and above at one of the participating universities were invited to take the online questionnaire. Those who were excluded from the study included non-medical undergraduate students, medical graduates who had already finished by the time the study commenced, and undergraduate medical students who declined to participate.

### Instruments

We used an online questionnaire containing three sections. The English version of the questionnaire was used in this study. The first consists of ten questions covering the sociodemographic aspects of the participating medical students. In the next section, we used the Survey of Attitudes Toward Statistics (SATS)-36 scale to assess students' attitudes towards statistics [10]. This scale was used in previous studies and showed good internal consistency and validity. SATS-36 scale comprises 36 items grouped under six different subscales: Affect: measuring students’ feelings concerning statistics (six items), Cognitive competence: measuring students’ attitudes about their intellectual knowledge and skills when applied to statistics (six items), Value: measuring students’ attitudes about the usefulness, relevance, and worth of statistics in personal and professional life (nine items), Difficulty: measuring students’ attitudes about the difficulty of statistics as a subject (seven items), Interest: measuring students’ level of individual interest in statistics (four items), and Effort: measuring amount of work the student expends to learn statistics (four items). A 7-point Likert Scale, ranging from 1 (strongly disagree) through 4 (neutral) to 7 (strongly agree), was used to capture the best sentiment of the respondent to each of these items. Responses of questions were reversed for negatively items (1 becomes 7, 2 becomes 6, 3 becomes 5, 5 becomes 3, 6 becomes 2, and 7 becomes 1). Total overall score and scores for subscales of SATS-36 ranged from 1 to 7. The last section, specifically, targeted students who used statistical analysis software -only been answered by participants who used statistical software-. It consisted of three questions to identify: the type of statistical software, statistical test or procedure used during data analysis, and the output format of presenting the data. All of these questions were multiple responses questions.

### Data collection and sampling

The online questionnaire was distributed anonymously across ten universities in Sudan. To ensure maximal coverage, 2–4 students from each university were assigned to distribute the survey link to their fellow students via various social media platforms as well as through personalized messages. Periodic reminders were sent to encourage more students to participate. The questionnaire was self-explanatory, and the responses were completely anonymized.

### Statistical analysis

Descriptive statistics for this data was presented as mean ± Standard deviation (SD) and number (percentage). To find if there is a significance difference between groups who are using or not using statistical analysis software, we used Mann–Whitney U test and independent t-test. A Univariate logistic regression was used to find the associated factors for using statistical analysis software. Data were analyzed using R software version 4.0.2.

## Results

### Sample characteristics

The overall response rate for all eligible participants in this study was 4.6% (489/10572). A total of 489 undergraduate students studying between 2^nd^ and 6^th^ year participated. The calculated mean (SD) age was 21.94 ± 1.61 years, with females representing just above half (52.4%, *n* = 256) of participating students. Of all participants, 228 (46.6%) reported attending a physical or online course in biostatistics, 144 (29.4%) studied the course of evidence-based medicine, and 175 (35.8%) had participated in a research project. However, only a quarter of the students (25.9%, *n* = 127) reported using a statistical analysis software (Table 1).

**Table 1:** Demographic data for undergraduate medical students according to the usage of statistical analysis software. (*n* = 489)

**Variables**	**Overall**, *N* = 489^1^	**Used Statistical analysis software**		***p*-value**^2^
		**Yes**, *N* = 127	**No**, *N* = 362	
**Age, Mean ± SD**	21.94 ± 1.61	22.34 ± 1.60	21.80 ± 1.59	**0.001**
**Gender, n (%)**				0.76
Female	256 (52.4)	65 (51.2)	191 (52.8)	
Male	233 (47.6)	62 (48.8)	171 (47.2)	
**Marital status, n (%)**				> 0.99
Married	13 (2.7)	3 (2.4)	10 (2.8)	
Single	475 (97.1)	124 (97.6)	351 (97.0)	
Widow	1 (0.2)	0 (0.0)	1 (0.3)	
**Year of study, n (%)**				** < 0.001**
Second	120 (24.5)	23 (18.1)	97 (26.8)	
Third	136 (27.8)	22 (17.3)	114 (31.5)	
Fourth	102 (20.9)	28 (22.0)	74 (20.4)	
Fifth	103 (21.1)	33 (26.0)	70 (19.3)	
Sixth	28 (5.7)	21 (16.5)	7 (1.9)	
**Received or attended any physical or online course in biostatistics, n (%)**	228 (46.6)	74 (58.3)	154 (42.5)	**0.002**
**Received or attended any physical or online course in Evidence based medicine, n (%)**	144 (29.4)	45 (35.4)	99 (27.3)	0.085
**Received or attended any physical or online course in Research methodology, n (%)**	284 (58.1)	99 (78.0)	185 (51.1)	** < 0.001**
**Having a family member (parent, sibling, spouse,..etc.)working in health care, n (%)**	262 (53.6)	74 (58.3)	188 (51.9)	0.22
**Participated in or establish a research project, n (%)**	175 (35.8)	78 (61.4)	97 (26.8)	** < 0.001**
**Published a research, n (%)**	28 (5.7)	20 (15.7)	8 (2.2)	** < 0.001**

^1^Data were presented as n (%); Mean ± SD

^2^Two Sample t-test; Pearson's Chi-squared test; Fisher's exact test

### Attitude toward statistics

The overall mean (SD) attitude toward statistics was 4.64 ± 0.91 in all participants. There was a significant difference in overall attitude score toward statistics showing a higher score for those who have used a statistical analysis software (4.86 ± 0.89 vs 4.56 ± 0.90, *p* = 0.001). In tandem with this, the mean score for each component of the students’ attitude was higher for those with prior experience at using statistical analysis software and the data also showed significant difference in affect (*p* = 0.002), cognitive competence (*p* = 0.002), value (*p* = 0.002), interest (*p* = 0.004) and effort (*p* = 0.029) (Table 2).

**Table 2:** Attitude towards statistics for undergraduate medical students (*n* = 489)

**Variables**	**Overall**, *N* = 489^1^	**Used Statistical analysis software**		***p*-value**^2^
		**Yes**, *N* = 127^1^	**No**, *N* = 362^1^	
**Overall attitude, Mean ± SD**	4.64 ± 0.91	4.86 ± 0.89	4.56 ± 0.90	**0.001**
**Affect mean, Mean ± SD**	4.46 ± 1.17	4.73 ± 1.20	4.37 ± 1.14	**0.008**
**Cognitive Competence mean, Mean ± SD**	5.13 ± 1.12	5.41 ± 1.08	5.03 ± 1.12	**0.002**
**Value mean, Mean ± SD**	5.13 ± 0.99	5.36 ± 1.02	5.05 ± 0.97	**0.002**
**Difficulty mean, Mean ± SD**	3.58 ± 0.90	3.50 ± 0.92	3.61 ± 0.89	0.25
**Interest mean, Mean ± SD**	4.86 ± 1.89	5.24 ± 1.82	4.73 ± 1.90	**0.004**
**Effort mean, Mean ± SD**	4.66 ± 1.82	4.94 ± 1.80	4.56 ± 1.82	**0.029**

^1^Data were presented as Mean ± SD

^2^Mann-Whitney U test test

### Usage of statistical analysis software

Overall, the use of statistical analysis software was very low among the students with only 25.9% reported previous experience with such software. Among them, the majority (71.7%) disclosed using the Statistical Package for the Social Sciences (SPSS) as the main software for analysis, while almost half of the students (50.4%) reported using Excel.

When it comes to statistical test preference, we found that 37.8% (*n* = 48) have used linear regression whereas 23.6% (*n* = 30) applied logistic regression in their analysis. Moreover, the utilization of Chi-squared test, Fisher exact test or McNemar test for categorical data was limited to a mere 26.8% of students (*n* = 34).

The most preferred format for presenting the data was tables utilized by 74.8% of the students (*n* = 95). This was followed by line chart/graph and histogram making up 25.2% (*n* = 32) and 24.4% (*n* = 31), respectively (Table 3).

**Table 3:** Types of statistical analysis software, statistical procedure and form for presenting data for undergraduate medical students who are using statistical analysis software (*n* = 127)

**Variables**	**Overall, N = 127**^1^
**Which statistical software programs you are using? (You can choose more than one response), n (%)**	
Statistical Package for the Social Sciences (SPSS)	91 (71.7)
Excel	64 (50.4)
R	3 (2.4)
Stata	8 (6.3)
Minitab	2 (1.6)
GraphPad Prism	3 (2.4)
Other (SAS, Chimera or Google Form)	3 (2.4)
**Which of the following statistical procedure you are using during analysis? (You can choose more than one response), n (%)**	
Linear regression	48 (37.8)
Logistic regression	30 (23.6)
Log rank test	7 (5.5)
Cox regression	5 (3.9)
Chi-squared test, Fisher exact test or McNemar test	34 (26.8)
Student's t-test, Mann–Whitney U test, Paired t-test or Wilcoxon signed-rank test	20 (15.7)
one-way analysis of variance (ANOVA) test, Kruskal–Wallis test, Repeated measures ANOVA or Friedman test	13 (10.2)
Correlation using Pearson's correlation (r), Spearman's (p), or Spearman/Kappa (Agreement)	18 (14.2)
**In which form you are presenting your data? (You can choose more than one response), n (%)**	
Table	95 (74.8)
Histogram	31 (24.4)
Frequency polygon	9 (7.1)
Frequency curve	16 (12.6)
Line chart/ graph	32 (25.2)
Cumulative frequency diagram	8 (6.3)
Scatter/ Dot diagram	10 (7.9)
Bar diagram	26 (20.5)
Pie/ Sector diagram	20 (15.7)
Pictogram/ Picture diagram	6 (4.7)
Map diagram/Spot Map	2 (1.6)
Box plot	1 (0.8)

^1^Data were presented as n (%)

### Associated factors for using statistical analysis software

On applying the univariate logistic regression, we found a significant association between being a final year medical student compared with 2^nd^ year medical student and the usage of analysis software (OR: 12.652, CI 95% 4.803– 33.332; *p* < 0.001). Likewise, using various analysis software was found to be significantly associated with older age (OR: 1.224, CI 95% 1.079– 1.388; *p* = 0.002), receiving physical or online courses in biostatistics (OR: 1.886, CI 95% 1.252– 2.841; *P* = 0.002) or research methodology (OR: 3.383, CI 95% 2.120– 5.398; *p* < 0.001), initiating or participating in a research project (OR:4.349, CI 95% 2.839 – 6.661; *p* < 0.001) or publishing research paper (OR: 8.271, CI 95% 3.542 – 19.312; *p* < 0.001) (Table 4).

**Table 4:** Univariate logistic regression for associated factors of using statistical analysis software

**Variables**	**Univariate Logistic regression**			
	**OR**	**95% CI**		***p*-value**
		**Lower**	**Upper**	
**Age**	1.224	1.079	1.388	**0.002**
**Gender**				
Female	-	-	-	-
Male	1.065	0.711	1.597	0.759
**Year of study**				
Second	-	-	-	-
Third	0.814	0.427	1.550	0.531
Fourth	1.596	0.851	2.993	0.145
Fifth	1.988	1.075	3.677	0.028
Sixth	12.652	4.803	33.332	** < 0.001**
**Received or attended any physical or online course in biostatistics**	1.886	1.252	2.841	**0.002**
**Received or attended any physical or online course in Evidence based medicine**	1.458	0.948	2.243	0.086
**Received or attended any physical or online course in Research methodology**	3.383	2.120	5.398	** < 0.001**
**Having a family member (parent, sibling, spouse…etc.) working in health care**	1.292	0.859	1.944	0.219
**Participated in or established a research project**	4.349	2.839	6.661	** < 0.001**
**Published research**	8.271	3.542	19.312	** < 0.001**

*CI* Confidence Interval, *OR* Odd ratio

## Discussion

Up to our knowledge, this is the first study ever to assess the attitude towards statistics and associated factors for using statistical analysis software among Sudanese medical students. Our final results showed an average attitude towards statistics with a higher attitude recorded among undergraduate medical students who had prior experience with statistical analysis software. SPSS and excel were the most commonly used statistical analysis software. Furthermore, being a final year medical student compared with second year medical student, publishing or participating in a research study, and attending research methodology courses were associated factors for using statistical analysis software.

Understanding statistics is essential especially for undergraduate medical students to help them critique clinical research and perform statistical analysis independently. Some studies highlighted several reasons contributing to medical students’ poor interest in biostatistics [11]. Recently, a study from China reported an improvement in the perception of statistics among postgraduate medical students after implementing block curriculum design [3]. Our study reported an average overall attitude towards statistics, and using an implementation in the curriculum would enhance the attitude among undergraduate medical students.

Previously, attitude toward statistics has been measured in several studies using a survey of attitudes towards statistics (SATS) developed by Schau [12]. In this study, the overall attitude toward statistics was mostly positive and above average, and the highest score attained for components of SATS was 5.13 ± 1.12 for Cognitive Competence. A similar study used the same tool reached a mean attitude score toward statistics of 4.41 ± 0.68 [13] which is slightly lower than our study. Interestingly, a study that had investigated the same topic but among student nurses found a negative correlations between the SATS score and with college level education, high school rank, exposure to statistics in high school and previous exposure to statistics in college [14]. Which means that been with these factors would results in low attitude toward statistics.

Engagement of student in biostatics course would improve their attitude toward statistics, and this fact was confirmed by researchers from Greece found that a biostatistics course significantly improved the attitude toward statistics among nursing students [15]. This means that we can apply a similar biostatistics course and consequently, it will lead to an improvement in the attitude toward statistics among undergraduate medical students.

This study also investigated the usage of statistical analysis softwares. Our results highlighted limited experience in using statistical analysis softwares by participating students. That’s being said, SPSS and excel were the two most commonly used statistical software programs for data analysis purposes. This corresponds with the findings of a previous bibliometric study identified SPSS as the most widely used statistical software program for statistical analysis of previously published research [16]. On the contrary, in the United States, Stata and SAS were the most frequently used statistical software programs for analyzing previous published health services research [17].

Among those with prior experience with analysis software, tables were the most preferred form for presenting the data. No previous study sought to identify the associated factors of using statistical analysis software among medical students. In our study, we found that being an undergraduate medical student in the sixth year, attending physical or online course in research methodology, initiating or participating in a research project and publishing own research are associated with higher usage of statistical analysis software. Therefore, by focusing on strengthening these associated factors, the percentage of users of statistical analysis software can be increased by inviting more students to indulge in research which will ultimately result in better attitude toward statistics and improved overall learning experience.

This cross-sectional study is considered the first to attempt to determine -on a large scale- the associated factors for using statistical analysis software among medical students from several universities in Sudan. By identifying these factors, this will help researchers and universities focus mainly on improving these associated factors and ultimately improving students’ overall attitude towards statistics. Also, it will improve the quality of medical education in undergraduate medical students.

Unfortunately, this study faced some limitations. To begin with, the online survey was distributed among medical students using convenient sampling technique and low response rate due to the shutdown of universities during the coronavirus-19 (COVID-19) pandemic. Secondly, the responses from medical students varied according to the year of study which gives unequal responses from each batch. Lastly, due to COVID-19, this study was conducted using an online survey; therefore, some students who did not have internet access could have been unintentionally excluded from participating in the study.

## Conclusion

Our study showed an average attitude toward statistics and low usage of statistical software among all participants. This level of attitude toward statistics was significantly higher among undergraduate medical students who had used statistical analysis software. Being a final year medical student, publishing or participating in research activity, and attending research methodology courses were identified as associated factors for using statistical analysis software.

Students should participate in research to increase their usage of statistical software; because they need statistical software during analyzing their data. Also, supplementary courses in statistics should be implemented in order to increase the prevalence of students who use statistical software and their attitude toward statistics.

## Data Availability

The data of this study are available from the corresponding author on reasonable request.
